# Hemophilia B gene therapy: a NAb–solute barrier overcome by adeno-associated virus serotype 5

**DOI:** 10.1016/j.rpth.2026.103428

**Published:** 2026-03-25

**Authors:** Radoslaw Kaczmarek

**Affiliations:** Herman B Wells Center for Pediatric Research, Department of Pediatrics, Indiana University School of Medicine, Indiana, USA

Early preclinical and clinical studies on gene therapy using adeno-associated viral (AAV) vectors established that neutralizing anti-AAV antibodies (NAbs) block transduction, thereby abolishing efficacy. In mice and nonhuman primates, coinjection of AAV8 with immunoglobulins from an anti-AAV8–positive individuals, or pre-existing titers exceeding 1:5, prevented transgene expression [1,2]. Similarly, phase 1/2 clinical trials of gene therapy for hemophilia B showed that anti-AAV2 and anti-AAV8 titers as low as 1:1 to 1:3.8 reduced factor (F)IX expression by 50% to 96% [3,4]. These findings led to the universal exclusion of gene therapy candidates with pre-existing NAbs, regardless of vector serotype.

This paradigm shifted when AMT-060 (AAV5-FIX) phase 1/2 investigators found that 2 vector recipients who originally tested negative for anti-AAV5 NAbs and showed meaningful therapeutic responses (1.3 and 6.8 IU/dL) displayed surprisingly high anti-AAV5 titers (1:87 and 1:256) when reevaluated with a total antibody (TAb) assay. Subsequent testing with a more sensitive NAb assay revealed even higher titers (1:210 and 1:340), and additionally, 1 more serum sample tested positive (1:21; FIX coagulation activity 3.0 IU/dL). These individuals would have been excluded had the more sensitive assay been used at screening. These results prompted a phase 2b study of the same vector (but now encoding the FIX Padua variant), which dosed 3 individuals with known pre-existing anti-AAV5 NAbs, all of whom achieved robust therapeutic responses (FIX coagulation activity, 33.2-57.0 IU/dL) [5]. Consequently, HOPE-B became the first phase 3 hemophilia trial to intentionally include NAb-positive candidates (21 of 54 recruited individuals), and NAb-positive people with hemophilia are now eligible for the approved product [6,7]. The 4-year follow-up efficacy and safety outcomes in this NAb-positive subgroup of the HOPE-B cohort are reported in this issue by Klamroth et al. [8].

Both anti-AAV5 positive (*n* = 21) and negative (*n* = 33) participants received 2 × 10^13^ vg/kg of etranacogene dezaparvovec, except for 1 NAb-positive individual who received one-tenth of the dose due to an infusion-related reaction. At year 4, the mean FIX level in NAb-positive responders was 34 IU/dL, similar to 37 IU/dL in the total HOPE-B cohort. The annualized bleeding rate decreased from 4.64 in the lead-in period to 0.58 in year 4, with continued reduction during months 7 to 48. One participant returned to prophylaxis during year 3 upon enigmatically losing FIX expression between months 29 and 30 after having had stable expression ranging from 9.6 to 12.9 IU/dL between months 7 and 24. Only 1 NAb-positive participant, who had the highest anti-AAV5 titer (1:3212), did not respond to etranacogene dezaparvovec. All other participants (titers up to 1:678) who received the full dose showed therapeutic response. Five of 7 infusion-related reactions in HOPE-B occurred in NAb-positive individuals. Otherwise, no consistent association between anti-AAV5 NAbs and safety was found.

The permissive NAb titer cutoff for etranacogene dezaparvovec remains unknown because anti-AAV5 titer did not significantly correlate with FIX levels within the permissive titer range in HOPE-B (up to 1:678). In nonhuman primates, the same NAb assay that was used in HOPE-B showed that anti-AAV5 titers as high as 1:1030 did not prevent transgene expression, but the assay conditions were different, which makes the cutoff untranslatable for the clinic [9]. An ongoing trial (NCT06003387) administering etranacogene dezaparvovec is recruiting 35 participants with hemophilia B and detectable AAV5 NAbs, including at least 10 with NAb titers of ≥1:1400 and will hopefully provide answers.

The mechanism of AAV5 transduction *in vivo* in the presence of NAbs remains unclear. AAV5 diverged from the common AAV clades, with capsid protein sequence only 51% to 59% homologous, compared with ∼85% homology between AAV2, AAV8, and AAVrh10 [10]. This low homology could make AAV5 less susceptible to crossreactive NAbs raised in response to other serotypes, which would bind AAV5 with lower affinity and avidity. Many individuals test positive for multiple AAV serotypes, with anti-AAV2 titers typically higher than all others. This suggests that they could have been exposed to AAV2 only and developed NAbs crossreacting with other serotypes, rather than having been infected with all those different AAVs [11,12]. In such scenarios, anti-AAV5 NAbs detected in etranacogene dezaparvovec trials could have been anti-AAV2 crossreacting with AAV5, with the crossreactivity being too weak to block transduction. High titer anti-AAV5 NAb would be more likely to represent a true history of AAV5 infection and prevent transduction. Validating this hypothesis in preclinical studies is difficult because animals develop more specific antibody responses to AAV than humans. Nevertheless, an early animal study comparing transduction efficiencies among AAV2, AAV5, and AAV8 found that AAV5 transduced nonhuman primate livers in the presence of circulating high titer anti-AAV2 antibodies or presumable low-titer anti-AAV5 [13].

Against this backdrop, clinical trials of valoctocogene roxaparvovec AAV5 gene therapy for hemophilia A used the same vector serotype and vector dose at the same order of magnitude (6 × 10^12^ to 6 × 10^13^ vg/kg) but excluded anti-AAV5 TAb–positive candidates, and the currently approved product comes with a companion diagnostic TAb assay to screen out TAb-positive individuals [14,15]. This discrepancy points to the lack of standardization and importance of assay conditions for the quality of results they produce. Both the ELISA-based TAb assay and cell culture–based transduction inhibition NAb assay (used for screening in the etranacogene dezaparvovec clinical program) are semiquantitative tests, measuring titers as the inverse of serum dilution; so, the higher the antibody titer, the higher the serum dilution. Neither assay has been standardized across the industry, and for NAb assays, full standardization may not be feasible due to differences between AAV vectors and their transduction efficiencies. Consequently, anti-AAV titers cannot be compared between clinical programs because the same serum samples could produce vastly different titers, even when using the same assay type but different parameters. For example, the original phase 1/2 trial of valoctocogene roxaparvovec screened candidates using an NAb assay with the multiplicity of infection (a number of reporter AAV vector particles per cell in a cell culture) of 25,000 [16], which is >66-fold higher than 378.4 in the AMT-060 titer re-evaluation study and >6-fold higher than in HOPE-B, meaning that the same serum sample (with all other assay conditions identical) would produce >66-fold or >6-fold higher titer in the etranacogene dezparvovec NAb assays, respectively, than that in the phase 1/2 valoctocogene roxaparvovec NAb assay [9,17]. For example, in the latter assay, the highest permissive NAb titer from HOPE-B trial (1:678) would only be ∼1:114 (Figure). These discrepancies show the need for caution when interpreting anti-AAV titers reported in clinical programs. A seemingly high titer may reflect assay sensitivity rather than the magnitude of the immunological barrier to transduction. Notably, 2 individuals in the pivotal phase 3 program of valoctocogene roxaparvovec seroconverted between screening and vector dosing (<1:20, 1:56, and 1:91 anti-AAV5 TAb titers) and showed therapeutic responses between FVIII:C 2.0 and 9.2 IU/dL at 152 to 156 weeks, supporting that pre-existing antibodies are at least partially permissive of transduction by AAV5 [18].

**Figure:**
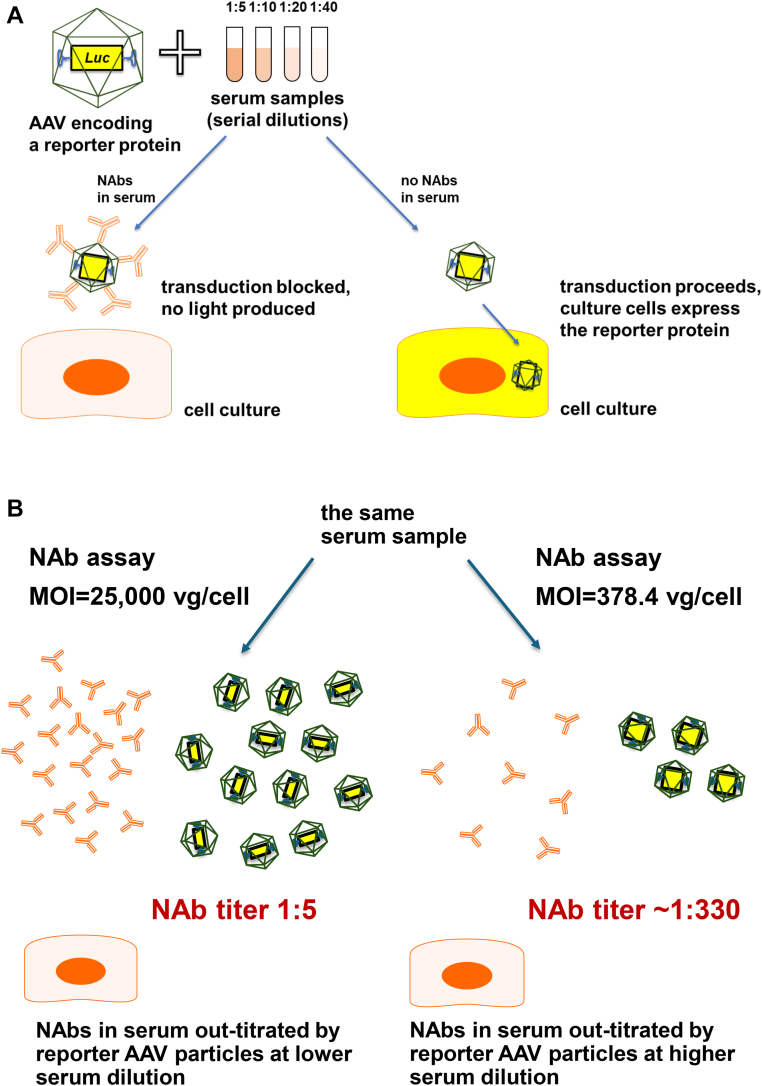
Anti-AAV neutralizing antibody assay principles. (A) Transduction inhibition or NAb is a semiquantitative assay measuring anti-AAV titers as the inverse of serum dilution. NAb assays use AAV vectors encoding reporter proteins. NAbs (if present in the tested serum) may block the vector from transducing the culture cells. Otherwise, the reporter AAV can transduce the cells and make them produce the reporter protein. Reporter proteins produce light, the amount of which can be measured. Assay parameters can have a profound impact on the quantification of NAbs, which can result in different rates of seropositivity and titers. Variation between assay parameters includes the cell type (different cell lines are variably permissive to transduction), serum volume, AAV serotype, reporter vector expression system, reporter signal reader, concentration and ratio of vector to cells (MOI), and empty capsid content. (B) A single assay parameter (in this example, the MOI) variation may have an outsized impact on the titering result. In an NAb assay, the MOI and the measured titer are inversely related. At a higher MOI, there are more viral particles for the same amount of antibodies to neutralize. Consequently, the transduction inhibition threshold is achieved at a lower serum sample dilution, resulting in a lower reported titer. If a hypothetical serum sample tested at an MOI of 25,000 vector genomes per cell (as in the phase 1/2 trial of valoctocogene roxaparvovec) showed a relatively low titer of 1:5, the same sample measured using a more sensitive assay at an MOI of 378.4 vector genomes per cell (as in the AMT-060 titer re-evaluation study) would result in a significantly higher titer of ∼1:330. This illustrates that titer cutoffs are not interchangeable across different clinical programs and reflect assay design rather than a true barrier to transduction. AAV, adeno-associated virus; MOI, multiplicity of infection; NAb, neutralizing antibody.

The ability of AAV5 to provide therapeutic effect in Nab- or TAb-positive individuals, NAb and TAb assay conundrums, the unexplained delayed loss of expression in 1 HOPE-B participant, and the apparent trend for higher incidence of infusion-related reactions in NAb-positive individuals are reminders of the many unknowns about AAV gene therapy. The delayed loss of FIX expression remains unclear, but it was unlikely related to the NAb status. The apparent efficacy NAb titer cutoff between 1:678 and 1:3212 necessitates NAb screening in candidates for the commercial product and informed decision because vector dosing elicits a strong NAb response that would preclude the vector recipient from availing of another gene therapy based on AAV in the future [19]. For the same reason, health care teams delivering AAV gene therapy need to be prepared to effectively manage infusion-related reactions and thus avoid underdosing. Importantly, multiple preclinical and early-stage clinical programs, including gene editing approaches, still rely on AAV to deliver transgenes [20]. Therefore, every attempt at providing gene therapy should be geared toward making the most of the opportunity to make it the coveted “one-and-done” intervention rather than “one-and-done-for.” HOPE-B has shown that it can be a one-and-done intervention for as many as one-third of individuals with hemophilia B regardless of their NAb status, highlighting the potential of gene therapy to overcome barriers to a functional cure, which is the goal of innovation in hemophilia treatment.
